# The Potential of Hydroponic Kit-Based Growing on a Self-Fertigation System for Pagoda Mustard (*Brassica narinosa* L) Production

**DOI:** 10.1155/2022/1984297

**Published:** 2022-11-15

**Authors:** Sophia Dwiratna, Kharistya Amaru, Muhammad Achirul Nanda

**Affiliations:** Department of Agricultural and Biosystem Engineering, Faculty of Agroindustrial Technology, Universitas Padjadjaran, Jatinangor, Bandung, West Java 45363, Indonesia

## Abstract

Agricultural land has been converted into settlements following the population growth in various parts of the country. The productivity of horticulture, particularly pagoda mustard (*Brassica narinosa* L), decreases with the narrowing of fields. The main milestone as a promising solution to overcoming this issue is applying the hydroponic technique. This study aims to analyze the potential of hydroponic kit-based growing on a self-fertigation system for pagoda mustard production. In contrast to general hydroponic, the proposed hydroponic kit is supported by a smart valve component as a unique novelty used for the automatic distribution of nutrients without electrical power (zero energy). The mustard seeds were sown on rockwool for two to three days in a dark room and placed in the sun for seventeen days. A total of 50 pagoda mustard seeds were arranged evenly on a self-fertigation system tray following a zig-zag planting pattern for forty days. The seed has the following morphological characteristics: average height of 22.88 cm, biomass width of 26.42 cm, root length of 23.4 cm, and weight of 241.5 g. Furthermore, the production requires a total fertigation consumption of 186 L (equal to 0.0935 L/plant day^−1^) with an actual crop coefficient between 0.01 and 0.54. The proposed system shows good performance for mustard growth with a uniformity value between 80 and 89%. Finally, hydroponic kit-based growing on a self-fertigation system can be applied in various areas to produce and maintain a sustainable food supply.

## 1. Introduction

Many countries rely heavily on mustards as a staple food in their diet. This is because the seed contains many polyphenolic compounds, including vitamins A, C, and E, calcium, and iron [1, 2]. Additionally, they provide an important source of dietary antioxidants and have high radical scavenging activity in preventing various chronic diseases such as cancer and cardiovascular disease [3, 4]. Based on the Central Bureau of Statistics [5], mustard production in Indonesia will reach approximately 667 thousand tons in 2020. The variety with a great economic value is the pagoda mustard (*Brassica narinosa* L), sold for IDR20,000 per 500 grams. Out of the 34 provinces in Indonesia, West, Central, and East Java are the top three producers of mustard.

However, the country's growing population has resulted in land conversion issues, directly narrowing agricultural land [6–8]. The narrower the land, the lower the horticulture productivity. The main milestone as a promising solution to overcoming this issue is applying the hydroponic technique. This is a cultivation technique using water or nutrient solution as a growing medium, known as soilless culture [9]. The main advantages are [10]as follows: (1) possible use of areas unsuitable for conventional agriculture, such as dry and degraded land; (2) plant independence for weather conditions; (3) year-round cultivation; and (4) reducing the use of labor-intensive activities such as weeding and soil preparation. The activity in hydroponics is also arguably lighter than conventional farming [11, 12]. Therefore, applying hydroponics to cultivate mustard is a solution to the massive conversion of agricultural land and increased productivity.

In hydroponics, water and nutrients are often supplied to plants using a fertigation system, which involves the injection of fertilizer into the irrigation stream. Some of the well-known systems include deep flow technique (DFT), nutrient flow technique (NFT), drip irrigation, Dutch bucket, and floating raft [13, 14]. However, the main drawbacks of these fertigation systems are (1) dependence on electricity throughout the cultivation of crops; (2) reasonably high investment and operational costs; (3) wasteful of water and nutrients; and (4) inefficient use of energy; and (5) unsuitable for underdeveloped regions. Therefore, this study proposes a breakthrough in environmentally friendly hydroponic technology based on a self-fertigation system, also well-known as Autopot [15–17]. Self-fertigation system provides nutrients automatically without using electricity (zero energy) throughout the cultivation. This advantage will lead to low investment and operational costs. As a result, it has grown rapidly in various regions in Malaysia [18], Australia [19, 20], and Saudi Arabia [17].

Hydroponic kits powered by self-fertigation systems are available with several trademarks, such as Autopot® Systems [18] and AQUAvalve5 [19], with a competitive price depending on the specifications. However, this research has designed the system supported by a smart valve component as a unique novelty used for automatic nutrient distribution compared to the type mentioned above. This is an improvement over previous technology, scientifically reported by Bafdal et al. [15]. The registered trademark for the system used is smart watering [20]. In the proposed self-fertigation, the smart valve component is designed to follow the principles of pressure and gravity. It signifies that the valve will open and close automatically based on the predetermined height, allowing the distribution and utilization of nutrients to operate efficiently and cost-effectively. This kind of technology is very suitable to be applied in areas that do not have access to electricity.

According to research reports, the self-fertigation technology effectively increases crop output by distributing nutrients precisely [15, 21–23]. Therefore, this system is suitable for horticultural commodities requiring large water and a relatively short harvest time. The implementation has covered various horticultural commodities such as cherry tomato, paprika, chili, cucumber, and wheat [15, 17, 21, 24]. However, no information about the application of mustard has been published to date. This research can also provide a new perspective on applying a self-watering system because each plant commodity has its characteristics. Therefore, this study aims to analyze the potential of a hydroponic kit based on the self-fertigation system for mustard pagoda production. This information is beneficial for various parties, especially growers, to effectively and efficiently run the production process.

## 2. Materials and Methods

### 2.1. Pagoda Mustard Cultivation

Pagoda mustard is one of the best vegetable plants because of its distinctive leaf form [25]. The seeds were obtained from Known You Seed Indonesia, Ltd. Pagoda mustard cultivation stages, including seeding, transplanting, and maintenance. First, the seeding process was carried out using rockwool planting media and placed in a dark room for two to three days. Second, the mustard seeds were removed and placed under the sun for seventeen days. True leaves are the first indication that the pagoda mustard is ready for transplantation.

**Figure 1:**
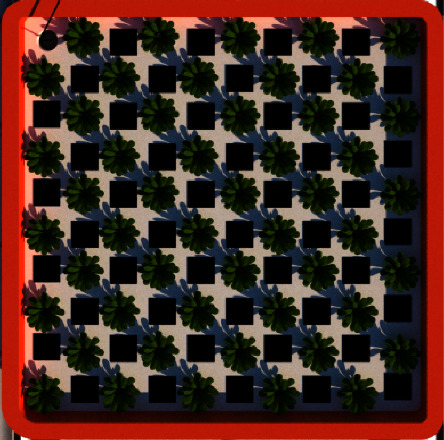
Pagoda mustard planting pattern.

The transplanting process was conducted from the seeding tray into the net pots and was transferred to the tray of the self-fertigation system until the harvest period. A total of 50 pagoda mustard seeds were placed evenly on the self-fertigation system tray following a zig-zag planting pattern (Figure 1). The planting distance was fixed at 20 cm since mustard has high biomass. Additionally, the roots of plants grown in net pots were permitted to protrude, allowing them to grow freely and be exposed to nutrients directly.

The next phase is maintenance, which tries to sustain plant life and suitable habitat. Daily maintenance of pagoda mustard addressed its water and nutrient needs. AB-mix fertilizer was utilized, combined with 5 L of water before applying. The nutrient solution was stirred until smooth to prevent the precipitation of fertilizer.

### 2.2. Rooftop Greenhouse

In this study, pagoda mustard was grown on the protected rooftop of a building at the Laboratory of Rooftop Urban Farming, Universitas Padjadjaran, Indonesia. It is located at coordinates 06° 55′ 23.4″ South Latitude and 107° 46′ 19.3″ East Longitude with an elevation of 794 masl. This location is suitable to support pagoda cultivation because they can produce well at an elevation of 250–1200 masl.

Rooftop cultivation is the right solution for urban areas to improve air quality and support food production [25]. Structurally, the rooftop greenhouse uses mild steel as the frame and transparent plastic as the roof. Therefore, the entire plant is well-shaded to create a humid microclimate in the crop area. Rooftop pagoda mustard gardening is anticipated to generate high-quality plants.

### 2.3. Self-Fertigation System

**Figure 2:**
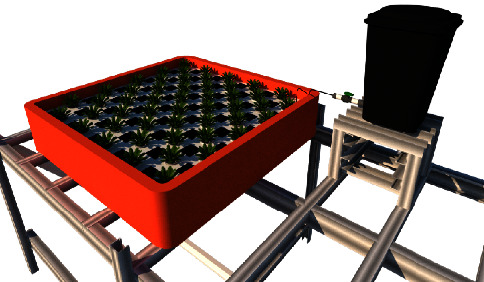
Self-fertigation system of pagoda mustard.

Water and nutrients in a hydroponic system can be carried out simultaneously using the principle of a self-fertigation system. The visualization is given in Figure 2. A plant tub is made of wood covered with a waterproof tarp with dimensions of 150 × 150 × 25 cm (l x *w* x *h*). This technique is comparable to floating raft hydroponics, with the primary distinction in the absence of circulating fertigation. In floating raft hydroponic, Styrofoam is used as a buffer to keep plants afloat on the nutrient solution. This self-fertigation system has 100 net pots with a diameter of 5 cm. However, this study only used 50 net pots due to the extensive pagoda mustard biomass consideration. Water and nutrients should always remain stagnant during the cultivation process in the planting tub. They were supplied with precision by a 50 L capacity reservoir supporting a vital component known as a smart valve.

**Figure 3:**
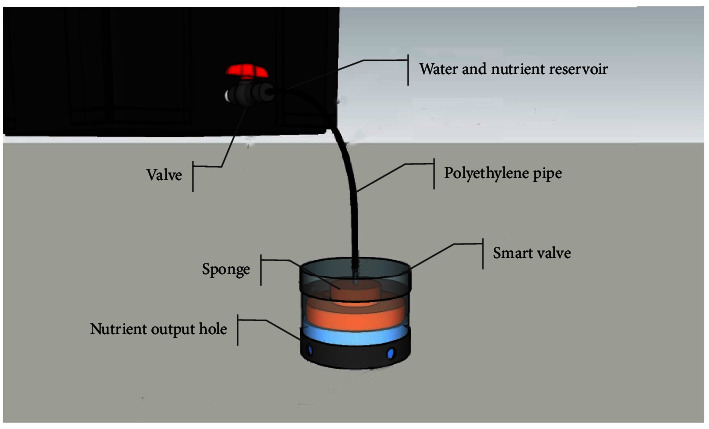
Smart valve components in the self-fertigation system.

A smart valve is a fertigation controller to set the maximum level of water and nutrients at a predetermined height. The float can move up and down following the water level in a tub. It opens and closes automatically based on the agreed initial setting. Furthermore, smart valves do not require electricity (zero electricity). Instead of using electrical energy, the smart valve is designed to follow the Archimedes principle. A buoyant force or upward thrust is applied to an object when partially or completely submerged in a fluid. Therefore, the construction size of each component should be calculated correctly. The smart valve can be seen in Figure 3, with each layer from top to bottom containing an input chamber, a sponge, and an output chamber.

The smart valve contains a sponge with dimensions of 6 cm in diameter and 4 cm in height for the automatic filling of water and nutrients. This implies that the nutrient reservoir will open automatically when the level in the planting tub is <6 cm and close at 6 cm. This sponge weighs less than the density of the liquid, hence the liquid exerts a buoyant force on it to a height of 6 cm. The smart valve works based on water pressure from the bottom, pressing up to close the flow of nutrients from the reservoir.

### 2.4. Observation Sample

The observation sample is calculated using the Slovin formula. This calculates the minimum sample size when the behavior of a population is not known with certainty. It is commonly used for survey research where the sample usually has a large size. Consequently, a formula is necessary to obtain a small sample representing the complete population. The number of samples using the Slovin approach can be calculated using equation (1), where *n* is the number of samples observed, *N* is the total population (50 net pots), and *e* is the error tolerance limit with a value of 10%.

(1) $$\begin{array}{c} n=\frac{N}{1+Ne^{2}}\text{.} \end{array}$$

**Figure 4:**
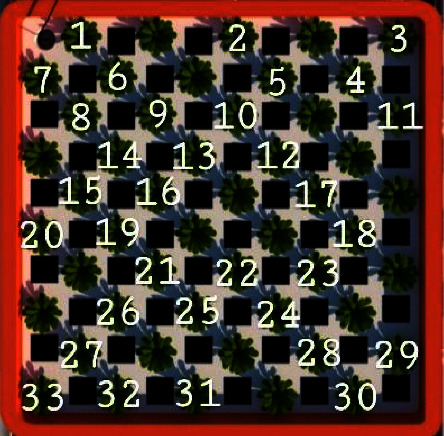
A total of 33 samples of pagoda mustard observed.

Based on the calculations, the minimum number of samples observed was 33. This was taken based on probability and simple random sampling techniques. Each population of mustard had an equal chance of being randomly selected to represent each row and column.  Figure 4 depicts the 33 samples of pagoda mustard that were analyzed.

### 2.5. Measurement

Measurements on applying a self-watering system for pagoda mustard are divided into microclimate, fertigation, and growth and yield. Each observation contains various measurement parameters to evaluate the performance of the proposed system. The procedure for each observation is discussed in detail below:

#### 2.5.1. Microclimate

Each measure should be precisely monitored as an evaluation indicator because the climatic conditions on the rooftop alter all the time. The parameters consisted of temperature, relative humidity (RH), light intensity, and wind speed. Microclimate measurements on the rooftop were recorded at 7.00, 12.00, and 17.00 daily during the pagoda mustard planting period. Calibrated instruments were used to provide accurate results, including a thermohygrometer for temperature and humidity, a lux meter for light intensity, and an anemometer for wind speed. The measuring instrument was placed in the appropriate location, and the result was displayed on the screen.

#### 2.5.2. Fertigation

Fertigation measurement includes evaluation of irrigation and nutrient during pagoda mustard cultivation, as discussed below:

*(1) Irrigation*. Water consumption (*W*_c_) is the amount of water used by plants. This is illustrated as the difference between the volume in the previous reservoir (*V*) and after it (*V*′). Water consumption can be calculated using equation (2). Furthermore, the water consumption of each plant on the *n*-th day after planting can be calculated by dividing the total consumption by the number of plants.

(2) $$\begin{array}{c} W_{c}=V-V^{\prime }\text{.} \end{array}$$

The next irrigation parameter is reference evapotranspiration calculated using the Penman-Monteith method [26]. This method requires some data, including temperature, humidity, duration of wind and sunlight, or available radiation. The Penman-Monteith method is recommended compared to others for the most satisfactory results [27]. In this study, the reference evapotranspiration value can be obtained using the CROPWRAT 8 software [28]. The Penman–Monteith method has the following equation:

(3) $$\begin{array}{c} ET_{o}=\frac{0.408\Delta \left(R_{n}-G\right)+\gamma \left(900/T+273\right)v_{w}\left(e_{s}-e_{a}\right)}{\Delta +\gamma \left(1+0.3v_{w}\right)}\text{.} \end{array}$$

The ET_o_ is the reference evapotranspiration (mm/day), *R*_*n*_ is the net radiation on the plant surface (MJ m^−2^ day^−1^), *G* is the density of the soil heat flux [MJ m^−2^ day^−1^], *T* is the daily air temperature at an altitude of 2 m [°C], *v*_*w*_ is the wind speed [m s^−1^], *e*_*s*_ is the saturation vapor pressure [kPa], *e*_*a*_ is the actual vapor pressure [kPa], Δ is the vapor pressure curve [kPa °C^−1^], and *γ* is a psychometric constant [kPa °C^−1^].

Crop evapotranspiration (ET_c_) is a parameter of water demand directly influenced by plant factors. It is related to crop water requirements, calculated based on the water consumption ratio on day-n to net pot area (*a*). Crop evapotranspiration can be calculated using Equation (4). Water requirements are affected by evapotranspiration and crop coefficient. Plants have different daily coefficients depending on the reference and crop evapotranspiration values [29]. The ratio is known as the crop coefficient parameter (*K*_c_) (Equation (5)).

(4) $$\begin{array}{c} ET_{c}=\frac{W_{c}}{a}, \end{array}$$

(5) $$\begin{array}{c} K_{c}=\frac{ET_{c}}{ET_{o}}\text{.} \end{array}$$

*(2) Nutrient*. Nutrient measurement parameters include solution temperature, electrical conductivity (EC), the potential of hydrogen (pH), and dissolved oxygen (DO). These measurements are recorded three times each day at 07.00, 12.00, and 17.00. This study executed temperature and EC parameters using the EC meter. Meanwhile, the parameters of pH and DO were measured using a pH and DO meter. Using these portable tools is very easy, and the operator only needs to dip the sensor probe into the nutrient solution to display the result on the screen. In addition, the requirement for nutrient solution during cultivation is determined based on changes in the reservoir's height.

#### 2.5.3. Growth and Yield

Measurement of growth and yield includes height, the width of biomass, root length, and biomass weight in pagoda mustard. The procedure for each parameter is described as follows: firstly, the height and width parameters measurement was conducted daily during plant cultivation until harvest using a ruler. The height measurement point started from the stem base in contact with the net pot to the highest leaf tip of pagoda mustard. Meanwhile, the width of the biomass was measured from the midpoint of the mustard greens to the farthest outer diameter. Secondly, root length and biomass weight parameters were measured at harvest (40 days after planting). The instruments used for measuring root length and weight were the rulers and analytical balance. Finally, biomass weight measurement was carried out directly after harvesting on a wet basis.

### 2.6. Data Analysis and Statistics

The performance of the self-fertigation system on the mustard pagoda was assessed using uniformity analysis to evaluate irrigation parameters, growth, biomass weight, and root length. The uniformity analysis metrics led to the success rate of an observation based on the percentage of uniformity coefficient (UC) values [31]. The higher the UC value, the better the performance of the self-fertigation system is [30].  Table 1 shows the UC value requirements based on uniformity analysis.

### 2.7. The Limitation of Study

This study focuses solely on the performance of the self-fertigation system in terms of microclimate, fertigation, and plant growth and yield. The proposed hydroponic kit was evaluated on a rooftop greenhouse during the rainy season in Indonesia's tropical climate. This evaluation is conducted from the period the plants are transplanted until harvest at 40 days. The results of this study are still limited to pagoda mustard cultivation. The value of microclimate, fertigation, and growth and yield may differ in other commodities because each plant has unique characteristics.

## 3. Results and Discussion

### 3.1. The Microclimate of a Rooftop Greenhouse

**Figure 5:**
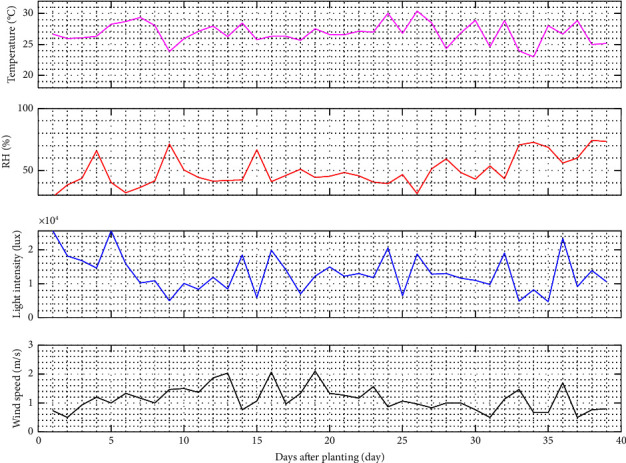
Microclimate conditions on the rooftop during pagoda mustard cultivation.

A study was conducted to examine the potential of hydroponic kit-based growing on the self-fertigation system. Pagoda mustard is grown on rooftops in a greenhouse to provide a high-quality product. Several microclimate parameters are monitored over time to ensure that these conditions are suitable for plant growth.  Figure 5 depicts the overall temperature, relative humidity (RH), light intensity, and wind speed during mustard pagoda cultivation.

Firstly, environmental temperature is essential in plant growth [31]. The inappropriateness can lead to morphological and physiological problems, where each plant has a unique environmental temperature range. Based on the analysis, the minimum and maximum temperatures in the rooftop greenhouse are 23–30.4°C. The temperature fluctuates every time, with an average of 26.87°C, and the maximum peak occurs 26 days after planting. The greenhouse temperature is directly proportional to plant transpiration. For example, the optimal temperature for pagoda mustard is 20–28°C. Mustard may adapt to extreme temperatures by consuming extra water and nutrients. The greenhouse temperature is directly proportional to the absorption of water and nutrients in plants.

Secondly, RH is a crucial factor in a greenhouse, defined as the proportion of atmospheric water vapor to the same temperature. It can vary according to the ambient temperature. Based on the analysis, the rooftop greenhouse has RH between 28.67 and 74.33% and an average value of 50.32%. The optimal for hydroponic cultivation is 70%. Furthermore, temperature and RH have an inverse relationship [32]. High RH can reduce the root's ability to absorb the nutrient solution.

Thirdly, plant growth is influenced by sunlight consisting of light intensity, quality (wavelength), and duration of irradiation (day length). It affects the opening of stomata, formation through chlorophyll and anthocyanins, absorption of nutrients, changes in leaf and stem temperature, transpiration, the permeability of cell walls, and protoplasm movement [33]. In this study, the intensity of sunlight in the greenhouse ranged from 4,686.67 to 25,584.0 lux, with an average of 12,969.6 lux. The light intensity is sufficient for the pagoda mustard because the greenhouse is located on the rooftop. In addition, it helps the plant to produce food from photosynthesis by absorbing water and carbon dioxide [34].

Plants can absorb visible light in photosynthesis, known as photosynthetic activity radiation (PAR) [35]. PAR is part of the solar radiation in the 400 to 700 nm wavelength range. It can be expressed in photosynthetic photon flux density (PPFD) as the amount of PAR of the light source in one unit of time [36]. The wavelength is used to quantify the intensity of sunlight, hence the bigger the PPFD, the greater the energy of each wavelength.

Lastly, wind speed is a microclimate factor affecting plant dryness and evapotranspiration. Plants can be harmed by excessive wind speed, yet it can be assisted by providing carbon dioxide. Based on the analysis, the greenhouse's minimum and maximum wind speeds are 0.5 and 2.1 m/s, respectively. The average wind speed is 1.13 m/s, and during the day, it is higher than in the morning and evening. The wind speed is directly proportional to evapotranspiration, thereby increasing water requirements for plants. Conversely, the wind that brings drier air will increase plants' transpiration [37].

### 3.2. Evaluation of the Self-Fertigation System

#### 3.2.1. Irrigation

**Figure 6:**
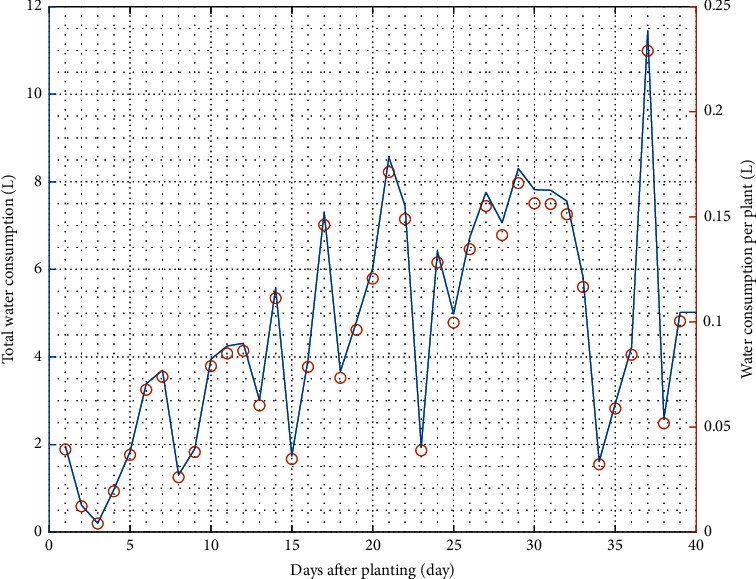
Water consumption during mustard pagoda cultivation.

Water consumption is the amount of water needed by plants in one planting period. It can be calculated based on the decrease in the reservoir before and after one planting period.  Figure 6 depicts the amount of water consumed during mustard pagoda cultivation. Based on the analysis, the total water consumption during one planting period was 183.73 L (equal to 0.092 L/plant day^−1^). The trend chart visually illustrates that pagoda mustards demand much water as they reach maturity and less when ready to be harvested (Figure 6). The extreme consumption occurred on day 36 after planting. This is related to microclimatic conditions that may not suit the mustard growth.

**Figure 7:**
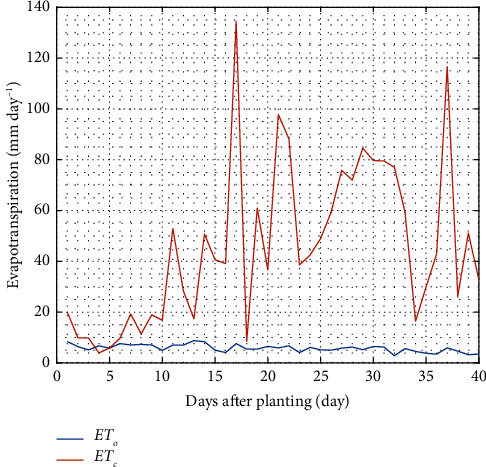
Fluctuations of reference (ET_o_) and crop (ET_c_) evapotranspiration.

Plant water requirement is the amount of water absorbed. It is influenced by evapotranspiration, plant growth phase, climatic factors, and plant types [16]. Reference evapotranspiration is the rate of evaporation in the microclimate. Therefore, the site selection for plant cultivation can determine the portion of water needed. The reference and crop evapotranspiration with crop coefficients was calculated on the consumption data. The reference and crop evapotranspiration can be seen in Figure 7.

The reference and crop evapotranspiration values have an increasing and decreasing trend every day respectively, leading to high and low water demand. The reference and crop evapotranspiration ranges from 2.86 to 8.8 mm/day and 3.93 to 134.49 mm/day. The 17 and 13 days after planting are the maximum peaks of reference and crop evapotranspiration, respectively. Evapotranspiration fluctuates greatly throughout the period due to the influence of various factors, including sunlight, temperature, RH, and wind speed. In general, reference evapotranspiration increases with these factors. Both reference and crop evapotranspiration are important parameters for calculating crop efficiency.

Another irrigation observation is the crop coefficient. This parameter can be influenced by climate, evaporation, plant type, and growth phase. For example, the *K*_c_ value for mustard is divided into the initial, development, midseason, and late-season stages for 17, 30, 35, and 45 days. During the initial phase, evapotranspiration is mainly in the form of soil evaporation [38].

**Figure 8:**
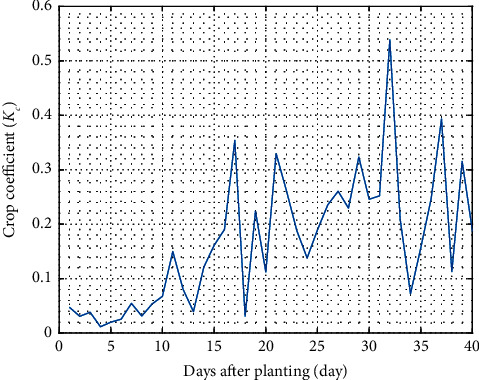
Crop coefficient during mustard pagoda cultivation.

Figure 8 depicts the actual crop coefficient values of pagoda mustard production's. Based on this figure, the actual crop coefficient ranges from 0.01–0.54. This is smaller than the theoretical (0.37–1.74) issued by FAO [38]. This is because cultivation is conducted using a fertigation system equipped with a protector (Styrofoam and nets pot) to minimize the effect of evaporation. As a result, the proposed system can save a high amount of water consumption. The plant growth stages consist of growing, vegetative and generative periods. The crop coefficient value for mustard in the early, middle, and late growth periods is 0.3, 1.2, and 0.6 [38]. Annual crops require much water throughout their growth. In contrast, perennial crops require less water because they have deep roots and are more drought-tolerant [39].

**Figure 9:**
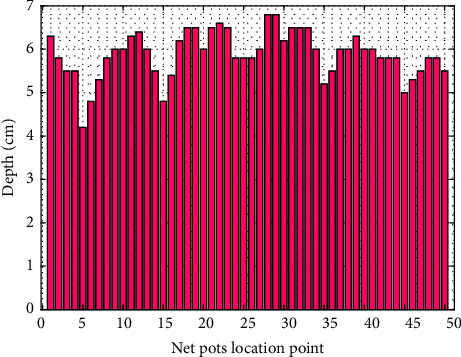
Water depth in the self-fertigation system.

The irrigation depth of the fertigation system can be seen in Figure 9. The water depth at the location of each net pot is relatively even, and in the self-fertigation system, it is 4.2–6.8 cm, with an average of 5.88 cm. Therefore, the irrigation uniformity is 92.99% based on numerical analysis, which indicates very good criteria. This uniformity provides advantages, such as a good irrigation process, effective nutrients, easy seedlings, good plant growth, and uniform harvest.

#### 3.2.2. Nutrient

**Figure 10:**
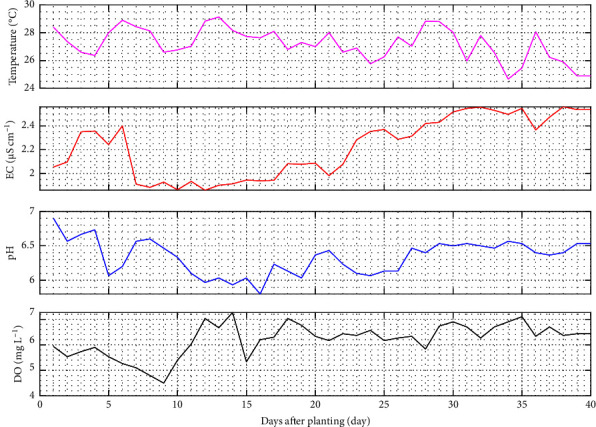
Nutrient parameters of the self-fertigation system during mustard pagoda cultivation.

Nutrient is the most vital factor in supporting good quality and yields. Therefore, providing nutrients that follow plant needs can achieve optimal growth and yield. Based on the analysis, the nutrient solution needed during mustard pagoda cultivation is 2.92 L (equal to 0.0015 L/plant day^−1^). Thus, fertigation consumption is 186 L (equal to 0.0935 L/plant day^−1^). Pada nutrient, this observation contains nutrient solution temperature, EC, pH, and DO parameters. These parameters will be discussed in detail in Figure 10.

Plants require optimal temperatures for growth and development. The recommended nutrient temperature for hydroponic plants is 18°C to 28°C. This study confirmed that the average nutrient temperature in the self-fertigation system was 27.19°C, with the minimum and maximum limits between 24.7 and 29.13°C, respectively. Based on these results, the nutrient temperature is still within safe limits to provide good plant growth. It is closely related to ambient temperature, which transfers heat from the nutrient solution. Plant development is impeded, and chemical processes are accelerated when the temperature of the nutrient is too high, leading plant physiological systems to be disrupted. Furthermore, increasing the temperature of the nutrient solution might reduce oxygen levels, limiting the roots' ability to absorb nutrients. Plants have more time to create cells, tissues, and organs when the nutrient temperature is low because chemical reactions take longer.

The next evaluation parameter on nutrient quality is EC. Based on the analysis, the self-fertigation system has an EC range between 1.86 and 2.56 *μ*S cm^−1^ with an average of 2.22 *μ*S cm^−1^. A good value for pagoda mustard is 1.6–2.5 *μ*S cm^−1^. As shown in Figure 10, EC experienced an increasing trend from 10 days after planting until harvest. Park and Cho [40] explained that changes influenced the trend in nutrient temperature, where the EC was inversely proportional to the temperature of the nutrient. The higher the temperature, the lower the EC, and vice versa. EC is closely related to the concentration of ions in the water, absorbed by the roots later. Therefore, the plant will absorb more nutrients when exposed to sunlight. The stomata are opened with the element Potassium (K^+^), causing the release of Hydrogen (H^+^) for the nutrient absorption process [41].

The next nutrient parameter is pH. Pagoda mustard has an optimal pH between 5.5 and 6.5. Based on observations, the self-fertigation system applied has an average pH of 6.34 for plant growth. However, some observations showed the pH was more than 6.5. This is caused by the high pH of the initial source of raw water. Therefore, 10% nitric acid (HNO_3_) was used to overcome this, and the pH in subsequent observations was under the criteria.

The last parameter in nutrients is DO. In cultivating aquatic culture plants, the area adjacent to the air has a higher DO and vice versa. This is because every plant cell needs oxygen to carry out respiration, whereas the roots will need the energy to absorb water and nutrients. The plant will die when its respiration is insufficient, and water and nutrients will no longer be absorbed. Therefore, adequate oxygen levels will improve the performance of the root in the speed of absorption of water and nutrients.

A root-in-water hydroponic system or water culture necessitates a DO range between 4 and 10 mg L^−1^. The nutrient solution in the hydroponic system is considered very good if the dissolved oxygen content is 8 mg L^−1^. Based on the measurements, the DO of nutrients in the self-fertigation system ranges from 4.1 to 7.9 mg L^−1^ and an average of 6.21 mg L^−1^. This confirms that the DO complies with the general recommendations that have been set for hydroponics.

### 3.3. Growth and Yield of the Mustard Pagoda

**Figure 11:**
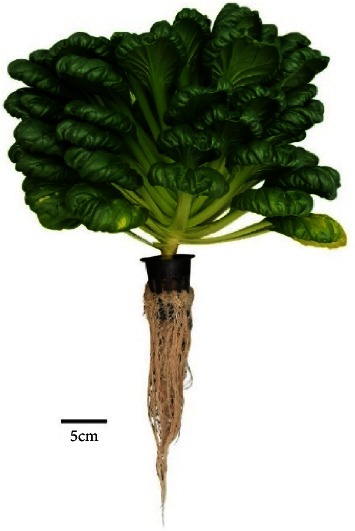
Pagoda mustard is ready to be harvested 40 days after planting.

The performance of the self-fertigation system was evaluated based on morphological measurements of the mustard pagoda. The parameters include height, the width of biomass, root length, and biomass weight. In this study, the pagoda mustard was measured in the vegetative stage. Therefore, morphological measurements were plant height and diameter. This is a morphological measurement technique to complete the mustard pagoda data's growth and yield, as shown in Figure 11.

**Figure 12:**
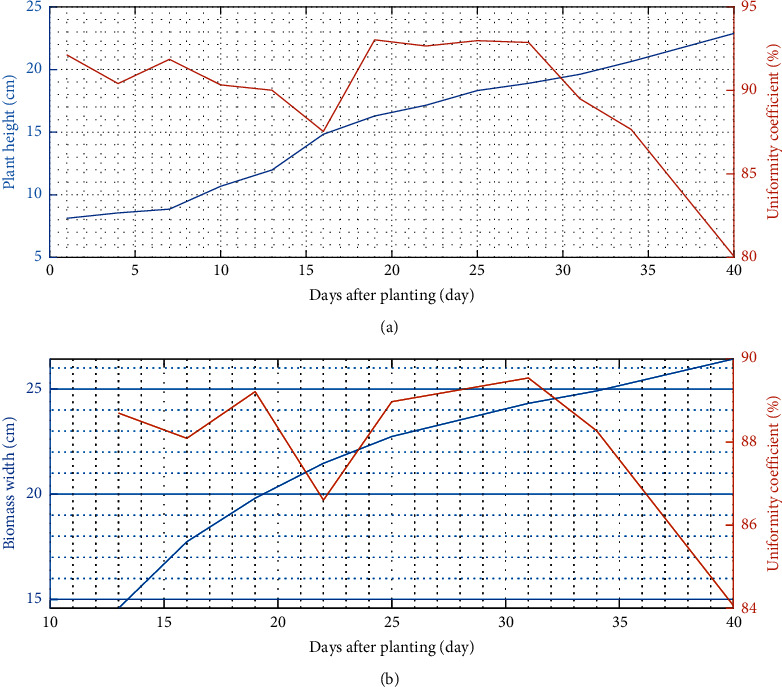
Pagoda mustard growth in the self-fertigation system: (a) plant height and (b) biomass width.

Figure 12 depicts the height and width of the pagoda mustard plant biomass. These parameters are growth indicators that are often observed to analyze the treatment response [42]. Based on the analysis, the average height and width of the pagoda mustard were 22.88 and 26.42 cm, respectively. In addition, the uniformity value for plant height and width was 80.07% and 84.07%. This implies that applying a self-fertigation system has good growth and development impact on pagoda mustard.

Leaf biomass is a plant vegetative organ with an essential role in photosynthesis. The biomass width affects the amount of sunlight needed for this process. Meanwhile, the biomass area is directly proportional to the amount of sunlight received. Nutrients are very influential in biomass formation, especially the element *N*, which plays a role in constructing shoots, leaves, and stems. A sufficient amount of *N* will impact the expansion of the leaf surface and lead to good yields [43].

**Figure 13:**
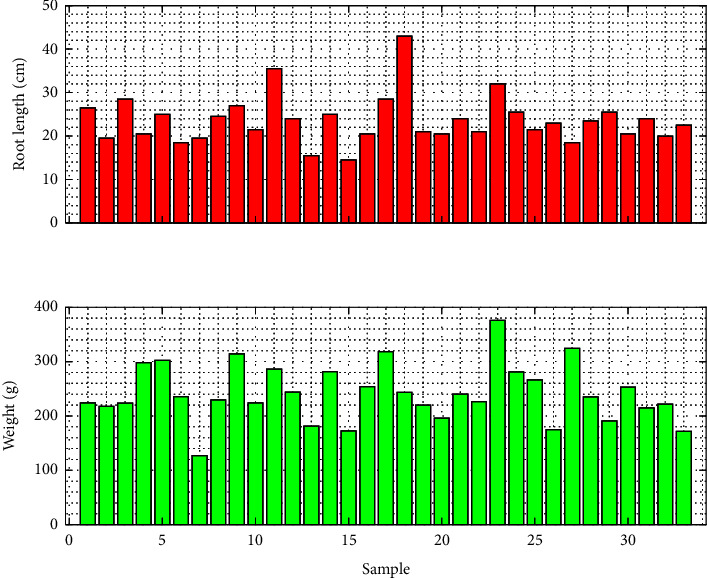
The weight and root length of pagoda mustard.

Several samples of pagoda mustard were harvested for yield measurement 40 days after planting. This measurement is derived from the plant growth process, where efficient photosynthesis produces optimal yields. Plant roots are one of the success factors for good growth because they are in direct contact with water and nutrient solutions. Based on the analysis, pagoda mustard has an average root length of 23.64 cm with a minimum and maximum length of 14.5 and 43 cm, respectively (Figure 13). Visually, the seed is white and has a straight and thick shape. Adequate oxygen levels regulate this to maintain its circulation in the water. Factors that affect the root distribution pattern include temperature, aeration, availability of water, and nutrients [41]. Therefore, good root growth will have an impact on increasing harvest weight.

Based on the analysis, pagoda mustard has an average plant weight of 241.5 g with a minimum and maximum value of 127 g and 376 g. Overall, the total product weight is 11.295 kg, with an area of 2.25 m^2^. Based on this data, land productivity in applying the self-fertigation system is 5.02 kg/m^2^. These results indicate that the self-fertigation system is considered suitable for mustard growth. Therefore, using a self-fertigation system can be a good solution to narrow agricultural land and increase the value of productivity. This research demonstrates that this system can be installed in various settings, including rooftops.

## 4. Discussion

In most metropolitan areas in developing countries, the existing system often fails to effectively meet the availability of horticultural products. Hydroponics offers a promising solution to deal with the scarcity of leafy vegetable supply. Also, hygienic, safe, and nutritious product quality is a major challenge at all levels of rural and urban social society. In this research, self-fertigation system-based hydroponic kit technology has great potential to overcome the above issues. This technology claims the slogan “zero electricity” to supply the availability of horticultural products while reducing operational production costs. Based on the performance evaluation, the hydroponic kit can adapt to the surrounding microclimate circumstances to maintain safe fertigation quality. Thus, plant growth can run optimally, leading to higher productivity.

The general factors for increasing productivity are creating suitable microclimate conditions, setting plant spacing, and availability of appropriate nutrients. In this study, the life cycle of pagoda mustard was concise compared to mustard grown traditionally on a soil-based basis in open fields, which could reach 60 days. One aspect of this accomplishment is the availability of nutrients during cultivation and directly to the root zone. In hydroponics, nutrients play an essential role in complementing the micronutrients and macronutrients. Nutrient distribution usually uses capillary action which works well for leafy vegetables, herbs, and spices. Pakpahan, HB, and Mahmudah [44] argue that the recommended concentration of nutrients for good growth of Pagoda mustard is 1400 ppm. Recently, several researchers have directed their studies in an environmentally friendly direction by using liquid organic fertilizers as alternative nutrients for hydroponics [45–47].

Evaporation in hydroponic farming is relatively low so this can be considered a water-saving method. Evapotranspiration as a major component of the hydrological cycle will influence crop water requirements and future planning related to irrigation management. Based on the analysis, this hydroponic kit has an evapotranspiration reference value of 2.86 to 8.8 mm/day, which has a fairly low evaporation rate. This will help the user since water and nutrients can be saved. Evapotranspiration in mustard has also been reported by previous researchers with various approaches, including 2.42 mm/day with Bowen's ratio energy balance approach [48], 132.6 mm/day by using the FAO dual crop coefficient approach [49], 7.98 mm/day by applying the FAO method [50], and 5.9 mm/day with FAO method [51]. The evapotranspiration value will correlate with the crop coefficient, representing crop and water management methods. If plants are often irrigated until harvest, the soil's surface will be wet and crop yield will be relatively high. Alternatively, the crop coefficient value will be quite low if the plants are allowed to dry on the land [49].

Growing plants with a hydroponic kit based on a self-fertilization system could be an appropriate technology. We hope this technology can be applied in underdeveloped areas in various developing countries where the scarcity of electrical energy is the main obstacle. There is also a lot of potential for this hydroponic kit technology to be used in places with low rainfall prone to drought. This hydroponic testing kit is not limited to only horticultural products, but we encourage various scientists to test on floricultural commodities. Thus, the characteristics of each commodity can be known so that the readiness level of hydroponic kit technology based on the self-fertigation system can run effectively and efficiently.

## 5. Conclusion

The results demonstrated that a hydroponic kit based on a self-fertigation system has good potential to be applied to pagoda mustard production. The proposed system can provide suitable conditions for plant growth during the cultivation process. Furthermore, the system is environmentally friendly because it requires no electricity (zero energy) throughout the cultivation of plants. Based on the analysis, pagoda mustard has the following morphological characteristics: average height of 22.88 cm, biomass width of 26.42 cm, root length of 23.4 cm, and weight of 241.5 g. The self-fertigation system showed good performance for growth with a uniformity value between 80 and89%. The production takes 40 days with total fertigation consumption of 186 L (equal to 0.0935 L/plant day^−1^). Therefore, hydroponic kits based on self-fertigation systems can be applied in urban and other areas without access to electricity to produce and maintain a sustainable food supply.

## Figures and Tables

**Table 1:** Uniformity criteria.

Criteria	UC value (%)
Excellent	>90
Good	80–89
Fair	70–79
Poor	<69

## Data Availability

The data used to support the findings of this study are available from the corresponding author upon request.
